# Decode protein-metabolite regulatory network: one MIDAS at a time

**DOI:** 10.1038/s41392-023-01566-6

**Published:** 2023-08-23

**Authors:** Tian Liu, Chen Gao

**Affiliations:** Department of Pharmacology and Systems Physiology, University of Cincinnati, Cincinnati, OH USA

**Keywords:** Biological models, Biochemistry

In a recent study published in *Science*, Hicks et al. utilized Mass spectrometry integrated with equilibrium dialysis for the discovery of allostery systematically (MIDAS) to explore protein-metabolite interactome and have revealed previously unknown regulation for lactate dehydrogenase.^1^ This report brings an exciting new approach to address a long-standing challenge in biology.

Cellular functions are orchestrated by an interactive network of molecular constituents, the physical interactions between proteins and other molecular entities, such as DNA, RNA, and metabolites. Like basic vocabulary for the book of life, these interactions are the fundamental mechanisms for complex physiology and diseases. Over the past two decades, the development of high-throughput sequencing and mass-spec based analysis tools helped to unlock the molecular identities and their interactions at unprecedented scales of complexity, yielding rapidly advanced understanding to protein-protein, protein-DNA and protein-RNA interactions. In contrast, the protein-metabolite interaction (PMI) remains poorly defined. Increasing evidence supports the importance of metabolites not just as substrates for metabolic reactions, but also as signaling molecules with potent functional impact through their interacting proteins. Therefore, developing unbiased approaches to systematically and comprehensively determine PMI network is critically needed to fill this important gap.

One recent development is LiP-SMap (Lip-small molecule interactions mapping) platform. It is a mass-spectrometry based technology taking advantage of altered protein sensitivity to proteolysis upon binding with metabolites. Whole cell lysates extracted under undenatured conditions are treated with or without a metabolite. Binding of the metabolite changes the proteolytic susceptibility of its cognate binding protein, allowing it to be differentiated from the non-binding proteins. Using LiP-SMap, Piazza et al. showed its potency by uncovering known and novel PMI interactions in *Escherichia coli* and revealed functional and structural principles of metabolite-protein communication.^2^ One unique feature of LiP-SMap approach is that it will not only map protein-metabolite interactions, but also provides information on the potential ligand-binding sites on the targeted proteins.

Another large-scale mapping tool is PROMIS (Protein-Metabolite Interactions) platform which combines size-exclusion chromatography with proteomic and metabolomic methods to detect interactions between untagged proteins and metabolites based on their co-segregation profiles. In a recent publication by Luzarowski et al., dividing yeast cells were used as a source for endogenous protein-protein and protein-metabolite complexes. The complexes were fractionated using size exclusion chromatography followed by liquid chromatography-mass spectrometry to detect both proteins and metabolites from the same fraction. A dataset for PMI based on co-segregation from chromatography was established for the entire proteome and metabolome in Saccharomyces cerevisiae.^3^ This report showcases the advantage of PROMIS for comprehensive profiling of PMIs in an unbiased fashion, however, the detection may be limited by the native abundance of the metabolites and their target proteins.

**Fig. 1:**
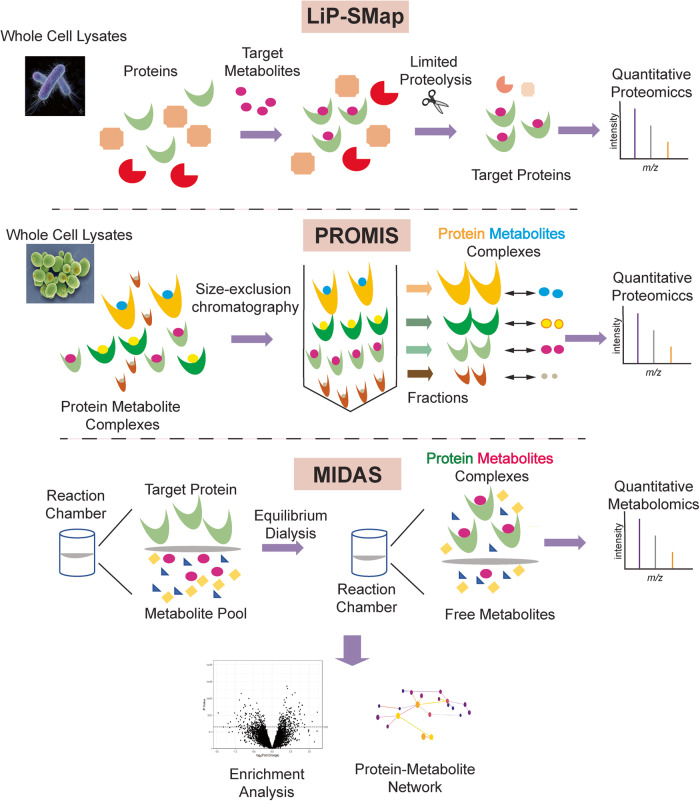
Overview of different protein-metabolite-interaction mapping methods: LiP-SMap (Lip-small molecule interactions mapping) utilizes altered protein sensitivity to proteolysis upon binding to metabolites to identify protein-metabolites interaction using the whole cell lysates. PROMIS (Protein-Metabolite Interactions) platform combines size exclusion chromatography with proteomic and metabolomic platforms to detect interactions between untagged proteins and metabolites using whole cell lysates. MIDAS(Mass spectrometry integrated with equilibrium dialysis) is a mass-spec based approach utilizing equilibrium dialysis of untagged metabolites. The purified target protein and a defined library of metabolites incubated in two chambers separated by a semi-permeable dialysis membrane that allows the diffusion of the metabolites

The most recent entry is MIDAS platform which is a mass-spec based approach utilizing equilibrium dialysis of untagged metabolites. This new technology has yielded the first detailed map of human PMI for specific metabolites and targeted proteins in carbohydrate metabolism, demonstrating a potential path to establish cellular PMI landscape in mammals.^1^ The concept of MIDAS is built upon on the biophysical principle of equilibrium dialysis (Fig. 1). This simple but effective design includes a purified target protein and a defined library of metabolites incubated in two chambers separated by a semi-permeable dialysis membrane that only allows the diffusion of metabolites. The non-interacting metabolites will reach equilibrium in both chambers at equal concentrations. However, the interacting metabolites will have higher or lower concentrations in the protein chamber depending on the binding affinity and mode of interaction. A positive fold change as measured by mass-spec in the protein chamber would indicate a direct PMI and a negative fold change can result from enzymatic conversion of the metabolite at a reaction rate faster than the diffusion rates. In principle, a comprehensive PMI can be established by exhausting the catalogue of proteins from any biological system.

To demonstrate the feasibility of MIDAS, Hicks et al. characterized a total of 33 human enzymes and 401 metabolites across major carbohydrate metabolic pathways, including glycolysis, gluconeogenesis, the tricarboxylic acid (TCA) cycle as well as serine biosynthetic pathway. A total of 830 putative PMIs were identified, including many previously unknown ones. Beyond these individual interactions, some global features begin to emerge. As expected, structurally and functionally related proteins frequently show similar metabolite interaction profiles, as demonstrated for lactate dehydrogenase (LDH) A and B. These two isoforms share common interacting metabolites including NADH and NAD. However, they also have distinct interaction profiles with two classes of metabolites, adenosine nucleotides and free or acylated CoA species. Using differential scanning fluorimetry (DSF) and enzyme activity assays, Hicks et al. validated that ATP inhibits only LDHA but not LDHB. In parallel, LDHA, but not LDHB, is also inhibited by fatty acid-acyl-CoA. Such differential interaction is also validated at functional level in intact cells. This discovery may have physiological importance as liver expresses mainly LDHA while the heart predominantly expresses LDHB. A differential PMI and metabolite-dependent inhibition for LDH activities may confer fuel utilization priorities between the two organs.

While this study represents a significant progress made in mapping human proteome interaction with metabolites, MIDAS has several limitations. Firstly, the current platform utilizes a cell-free system to measure PMIs in a defined condition. Therefore, the results may not capture the complexity of PMI in living cells, where sub-cellular compartmentation and protein-protein complexes may significantly alter the PMI profiles. Secondly, the interactions detected by MIDAS cannot infer directly for their functional outcome. The allosteric binding can be inhibitory or stimulative to a targeted protein, affecting its activity, stability, complex formation or intracellular localization. Painstaking efforts are required to further validate the functional outcome of such interactions. Thirdly, MIDAS can profile only relatively stable interactions while PMIs may be further expanded by the fact that many metabolites, including carbohydrates, lipid and amino acids, have dynamic flux in different tissues, requiring time-dependent interactions with targeted proteins.^4^ Lastly, MIDAS uses a defined environment in its assay, while PMIs are likely to be regulated by short or long-term stresses. Diseases like chronic heart failure and metabolic disorders will involve changes of proteins beyond expression levels, particularly in post-translational modifications.^5^ Despite these limitations, MIDAS may serve as another important tool to provide fundamental insights to decode the complex communications between proteins and metabolites in mammalian system. Like Rosetta stone to connect different languages, future efforts like MIDAS, will one day lead us to fully explore the communication between protein and metabolites in biology and diseases.
